# Inhibition of inflammation by minocycline improves heart failure and depression-like behaviour in rats after myocardial infarction

**DOI:** 10.1371/journal.pone.0217437

**Published:** 2019-06-24

**Authors:** Hong-Wei Wang, Monir Ahmad, Rami Jadayel, Fatimah Najjar, Diane Lagace, Frans H. H. Leenen

**Affiliations:** 1 Brain and Heart Research Group, University of Ottawa Heart Institute, Ottawa, Ontario, Canada; 2 Neuroscience Research Program, Department of Cellular and Molecular Medicine, University of Ottawa, Ottawa, Ontario, Canada; Max Delbruck Centrum fur Molekulare Medizin Berlin Buch, GERMANY

## Abstract

**Rationale:**

Patients with heart failure have an increased incidence of depression. Central and peripheral inflammation play a major role in the pathophysiology of both heart failure and depression.

**Aim:**

Minocycline is an antibiotic that inhibits microglia activation and release of pro-inflammatory cytokines. We assessed effects of minocycline on extent of heart failure and depression at 2 and 8 weeks post myocardial infarction.

**Methods/Results:**

Male Wistar rats were randomly divided into 3 groups: (i) sham + vehicle; (ii) MI + vehicle; and (iii) MI + minocycline with n/group of 8, 9 and 9 at 2 weeks, and 10, 16, 8 at weeks, respectively. Oral minocycline (50 mg/kg/day) or vehicle started 2 days before surgery. Depression-like behaviour was assessed with sucrose preference and forced swim tests, and cardiac function with echo and hemodynamics. After myocardial infarction, microglia activation and plasma/brain pro-inflammatory cytokines increased, which were mostly prevented by minocycline. At 8 weeks, cardiac dysfunction was attenuated by minocycline: infarct size (MI + Vehicle 29±1, MI + Min 23±1%), ejection fraction (Sham 80±1, MI + Vehicle 48±2, MI + Min 58±2%) and end diastolic pressure (Sham 3.2±0.3, MI + Vehicle 18.2±1.1, MI + Min 8.5±0.9 mm Hg). Depression-like behaviour was significantly improved by minocycline in sucrose preference test (% Sucrose Intake: Sham 96±1, MI + Vehicle 78±2, MI + Min 87±2) and forced swim test (% Immobile: Sham 40±4, MI + Vehicle 61±3, MI + Min 37±6).

**Conclusion:**

Rats post myocardial infarction develop systemic inflammation, heart failure and depression-like behaviour that are all attenuated by minocycline. Targeting (neuro) inflammation may represent new therapeutic strategy for patients with heart failure and depression.

## Introduction

Both heart failure (HF) and depression are very prevalent and major health concerns with tremendous consequences for quality of life, morbidity and mortality. There is accumulating evidence for extensive cross-talk between the cardiovascular system and the brain. HF is associated with a high incidence of depression, and patients with both HF and depression have a poorer quality of life, and > 2-fold increased risk of further cardiac events and mortality than those with HF but not depressed [1–4]. There are currently no effective treatments to reduce depressive symptoms in patients with HF. Recent randomized clinical trials concluded that treatment of these patients with a selective serotonin-reuptake inhibitor compared to placebo does not cause a significant improvement in depression symptoms and does not improve cardiovascular event rates [1–5]. There is, therefore, a critical need to better understand the mechanisms contributing to this adverse interaction in order to develop new effective preventative/therapeutic strategies.

The development of depression in patients with HF is not only due to psychosocial factors, but also is mediated by critical biological factors [6]. Indeed, animals with HF also develop depression like-behaviour [7–11]. Activation of the immune system in the brain (neuroinflammation) plays a major role in the pathophysiology of HF as well as depression [12–17]. Post myocardial infarction (MI), both circulating pro-inflammatory cytokines (PICs) and plasma angiotensin II (Ang II) increase [11,18] and can contribute to upregulation of Ang II-AT_1_R signaling and cytokines in the paraventricular nucleus (PVN) of the hypothalamus [14,15,17,19].

Activation of microglia cells, which are the resident innate immune cells in the CNS [20], plays a critical role in this signaling cascade. A chronic increase in plasma Ang II results in activation of microglia to secrete cytokines and increase reactive oxygen species (ROS) in the PVN [21–23]. Persistent activation of microglia also occurs in key cardiovascular regulatory nuclei such as the PVN and the rostroventrolateral medulla (RVLM) in the brainstem of rats with HF post MI [24]. Moreover, inhibition of this microglia activation by central or peripheral administration of the tetracycline, minocycline, prevents most of the Ang II- induced upregulation of cytokines in the PVN as well as the sympatho-excitation and hypertension, [22,23] and the neuronal activation in the PVN and RVLM of rats with HF post MI [25].

Microglia activation also occurs in animal models of stress-induced depression. For example, chronic unpredictable stress causes depression-like behaviour and microglia activation in the hippocampus and prefrontal cortex (PFC) [26,27]. Moreover, initiation of treatment with minocycline before the onset of stress prevents the stress-induced microglia and PICs activation [27] as well as depression-like behaviour in several models [28,29]. Marked increases in PICs such as IL-1β, IL-2, IL-6 and TNF-α also occur in the PFC of male rats with HF post MI [11].

Considering the above studies, we hypothesized that post MI microglia activation and increase in PICs in the PVN are critical for sympatho-excitation and progressive cardiac dysfunction, and in the PFC are critical for cytokine-induced activation of eg ROS and decrease in BDNF contributing to depression [13,30]. If so, chronic treatment with minocycline will inhibit both the development of HF post MI as well as the associated depression-like behaviour. To test this hypothesis, we assessed in male Wistar rats: 1) development of HF and depression-like behaviour after induction of MI, and 2) effects of short-term and long-term oral treatment with minocycline initiated before the MI on microglia activation, plasma and brain cytokines and development of HF and depression-like behaviour.

The results reveal that striking microglia and PICs activation and depression-like behaviour develop in the chronic phase of HF in rats post MI. Minocycline prevents increases in both plasma and brain PICs, and inhibits the development of HF as well as depression-like behaviour.

## Materials and methods

### Animals and surgeries

Male Wistar rats, 200–250 g at arrival from Charles River Canada (Montreal, PQ), were acclimatized in a room maintained at constant temperature and humidity. Animals were individually housed, kept on a 12:12-hr reversed light-dark cycle, with the dark cycle starting at 3:00 AM, and had access to standard laboratory chow and tap water ad libitum. MI was induced in rats pre-medicated with sustained release buprenorphine (1mg/kg, s.c.). Under general anaesthesia with 2% isoflurane in O_2_, thoracotomy was performed at the 4th intercostal space. The left anterior descending (LAD) coronary artery was permanently ligated 1–2 mm distal to its origin with a 6–0 silk suture on an atraumatic needle. Lungs were then inflated, air was removed from the thoracic cavity and the chest was closed with 2 separate 4.0 Vicryl sutures approximating 4th and 5th ribs together. Sham operated animals underwent the same procedure without LAD occlusion. All animal procedures were approved by the University of Ottawa Animal Care Committee and adhere to the standards set in the *Guide for the Care and Use of Laboratory Animals* published by the US National Institute of Health (NIH Publication, 8th edition, 2011). Only male rats were studied, since female rats post MI do not develop depression-like behaviour unless ovariectomized [11].

### Experimental protocols

#### Protocol 1: Depression-like behaviour post MI

An initial experiment was performed to establish the extent of depression-like behavior post MI. Rats were randomly divided into 2 groups: (i) sham-operated rats (n = 6); and (ii) MI rats (n = 10). Tests to detect depression-like behaviour were performed at 3–4 and 6–8 weeks post MI, and echocardiography and hemodynamic measurements at 8 weeks post MI. The 8 week results were similar to these found in the 8-week experiment of protocol 2, and data of the two experiments were combined.

#### Protocol 2: Effect of Minocycline for 2 and 8 weeks post MI

**Fig 1:**
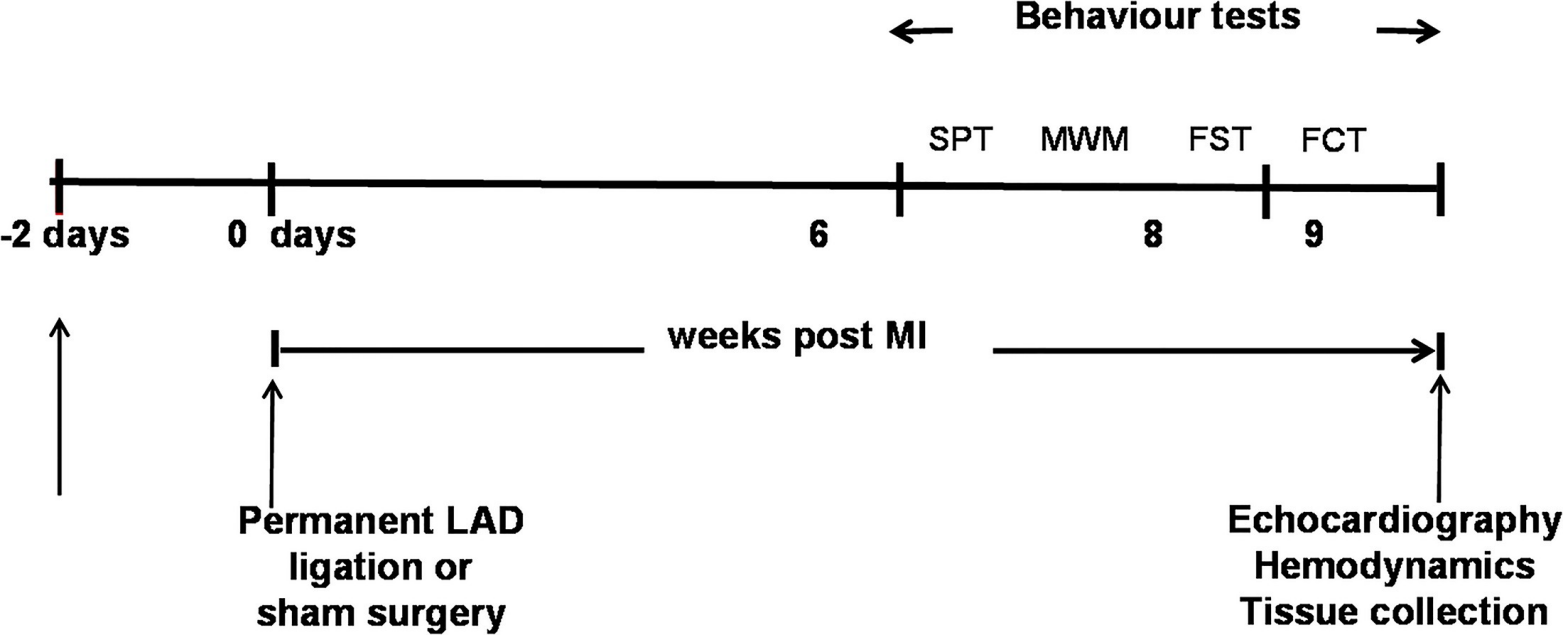
Timeline showing the experimental design for the 8 weeks post MI treatment study and the sequence of the behaviour tests. Male Wistar rats received orally daily minocycline or vehicle starting 2 days before MI surgery. Behavioural studies were carried out 6 to 8 weeks after surgery with first the sucrose preference test (SPT), followed by Morris Water Maze test (MWM), forced swim test (FST) and fear condition test (FCT). Except for the SPT, the behaviour tests were performed between 7:00 am and 2:00 pm in the dark cycle which started at 3:00 am.

Two days prior to surgery, rats were randomly divided into three groups: (i) sham rats receiving vehicle; (ii) MI rats receiving vehicle; and (iii) MI rats receiving minocycline. The MI surgery was performed blinded for the treatment allocation. A sham + minocycline group was not included because of limited time to study the behaviour of more animals during the dark cycle, and previous studies did not observe any effects of minocycline in normal animals [25, 31–33]. Oral dosing of minocycline (50 mg/kg/day) or vehicle (~ 5g peanut butter) began 2 days before surgery and continued for 2 or 8 weeks. This dose of minocycline is based on studies by Santisteban et al [22]. Peanut butter was pasted on the wall of the cage early in the morning starting 5 days before surgery to make the rats familiar to eat peanut butter for the drug treatment. All rats learned to eat the supply within 20–30 minutes. Tests for depression-like behavior (SPT and FST) were done at both 1–2 and 6–8 weeks post MI. Tests for cognitive function (MWM and FCT) were due to time-constraints only performed at 6–8 weeks post MI. After 2 or 8 weeks, echocardiography and hemodynamic measurements were performed, followed by blood and tissue collection to assess plasma and tissue cytokine levels or microglia activation. At the 2 week time point, the latter were also assessed in “naïve” rats (i.e. no intervention at all) to assess the impact of surgery per se [34]. Fig 1 shows the timeline for the 8 weeks study and the sequence of the behaviour tests.

**Behavioural Tests**. The sucrose preference test (SPT) and forced swim test (FST) are employed to test for anhedonia and behavioural despair, while the Morris water maze (MWM) is used to evaluate spatial learning and memory and the fear conditioning test (FCT) to test for learning and associative memory. Together, these behavioural tests are a powerful test battery for detecting both pro-depressant effects of stressors and anti-depression actions of drugs, as well as cognitive behavioural changes in rodents [35–37].

**Sucrose Preference Test (SPT)**: Rats were given concurrent access to a bottle with tap water and a bottle with 1% sucrose solution for one week. Positions of these bottles were switched every day to prevent any side preference. Sucrose preference was calculated as the percent of sucrose intake over total daily fluid intake, and is presented as the mean for the last 3 days.

**Forced Swim Test (FST)**: Each rat was placed in a translucent plastic cylinder (height: 45 cm, diameter: 20 cm) containing 30 cm of water at 25 ° C for 10 minutes. The total duration of immobility, defined by the absence of escape-oriented behaviour and displaying only passive movements necessary to keep its head above water, was determined using EthoVision XT 11.5 (Noldus Information Technology. Leesburg, VA, USA). Percent immobility was calculated as the percent of immobile behaviour over the test duration.

**Morris Water Maze (MWM)**: The water maze consists of a circular pool (diameter: 1.84 m) with a clear acrylic glass platform (diameter: 0.10 m) submerged in the center of the south west (SW) quadrant of the pool 1 cm below the surface of water at 25 °C. The maze was located in a dimly lit room with different black spatial cues mounted on the white wall in the south (S), west (W) and east (E) quadrant of the room. The MWM ran for 5 days with 2 training sessions on day 1 and 2, 1 probe session followed by a training session on day 3, 2 training sessions on day 4 and 1 probe session on day 5. Each training session consisted of 3 trials that had a inter-trial interval of ~ 10 minutes and an inter-session interval of 1 hour. For each trial the rat was placed into the pool in one of three designated release quadrants (NW, NE, SE) facing the pool in a random order, with the trial ending when the rat found the platform or after 1 minute trial duration. In order for the rat to orient with the spatial cues the rats remained on the platform for 15 seconds prior to being removed from the pool. The 1 minute probe trial was performed with the platform removed. During this test the rats were recorded and behaviors analyzed using the computer tracking software EthoVision XT 11.5. The learning curves for the training sessions and the times spent in the platform quadrant during the 2 probe sessions are presented.

**Fear Conditioning Test (FCT)**: Fear conditioning experiments were performed using the PhenoTyper cage and EthoVision 11.5 XT video tracking system. One Phenotyper cage with a shock grate floor and outside walls covered with board was used for acquisition (day 1) and testing context (day 2). Another Phenotyper cage was used for cue testing (day 3) that had different flooring and walls, as well as black insert inside the cage to both alter the dimensions of the cage and to put a 50 mL beaker of artificial vanilla extract hidden behind the insert in order to alter the scent of the cage. On day 1, rats were habituated for 2 minutes to the cage, followed by 2 tone-shock pairing that had a inter-trial interval of 2 minutes, followed by a 2 minute period in the absence of tone or shock. The tone-shock pairing consisted of playing of a tone (~2300 Hz, ~70 dB) for 30 seconds that ended concurrently with a 2 second foot shock (0.45 mA). On day 2, the rat was placed into the same cage as day 1 and then freezing behaviour associated with the context was recorded for 6 minutes. On day 3, rats first stayed for 3 minutes in the different cage without a tone, and then the tone was played for 3 minutes to assess freezing behaviour without and with the cue.

#### Echocardiography and hemodynamics

Using VisualSonics Vevo 770 System (VisualSonics, Toronto, ON, Canada), in M-mode recording, internal dimensions of the LV during systole and diastole were measured, and LV EF was calculated. Under isoflurane anaesthesia, a Millar catheter was inserted into the LV through the right carotid artery. This catheter was connected to a Harvard Data Acquisition System interfaced with a PC using AcqKnowledge 3.9 software. The level of anaesthesia was then gradually reduced until the animal started responding to a toe pinch. LV EDP, PSP, dP/dt_*min*_ and dP/dt_*max*_ were then recorded and measured for 30–60 seconds. Brains were removed and frozen in 2-methylbutane (-20 to -30° C) and stored at -80° C. Hearts were removed and placed in ice-cold saline. The LV was separated from the RV at the inter-ventricular septum, and was spread out on a transparent sheet. The LV infarct scar size was measured by planimetry and expressed as a percent of total LV area. Rats with MI scar size < 15% LV (28) were excluded from further analysis (at 2 weeks 3/11 of MI + veh and 3/12 of MI + min groups of Protocol 2, and at 8 weeks 3/10 of the MI group of Protocol 1, and 3/12 of MI + veh and 1/9 of MI + min groups).

#### Immunochemistry

Rats were euthanized with lethal dosage of sodium pentobarbital (65 mg/kg, IP) and transcardially perfused with ice cold ~250 mL PBS followed by cold ~300 mL 4% paraformaldehyde (PFA). Brains were post-fixed in 4% PFA at 4° C for at least 16 hours (up to 24 hours). The brains were cryoprotected in 30% sucrose/PBS at 4° C until used. Brains were frozen with OCT compound (VWR Clear Frozen Section Compound, VWR, Mississauga, ON, Canada) in a Peel-A-Way embedded mold (PolySciences Inc., Warrington, PA, USA). Twenty μm coronal brain cryosections were cut and mounted onto Superfrost Plus slides and stored at −80° C. The localization of the PVN was determined according to the rat atlas of Paxinos and Watson [38].

For immunostaining, sections were first dried in the fumehood, then washed with PBS, and blocked in 5% goat serum in 0.2% Triton-100/PBS for 1 hour. Sections were then incubated in microglia-specific rabbit anti Iba-1 primary antibody (1:800, Wako Chemicals USA, Inc. Richmond, VA, USA) at 4° C overnight, followed by incubation with Alexa Fluor 488-labelled goat anti-rabbit IgG secondary antibody (1:500, Invitrogen, Rockford, IL, USA) in 5% goat serum in 0.2% Triton X-100/PBS. Sections were mounted using Vectashield mounting medium with DAPI (Vector Laboratories, Burlingame, CA).

#### Analysis of microglia activation

Images were captured at 400× magnification using a Leica DMi8 fluorescence microscope equipped with a DFC9000 GT camera and Leica LAS X imaging and analysis software (Leica Microsystems Inc., Concord, Ontario, Canada). The images for the PVN were taken at -1.8 to -1.9 mm (posterior to bregma), and were analyzed for total microglia count and percent activation with ImageJ software. Activated microglia cells are defined as having an enlarged soma with stubby and shortened dendritic processes, and with an obvious increased expression of the microglia activation marker Iba-1. The number of microglia were counted in 4 squares of 0.2×0.2 mm^2^ with 2 squares placed laterally ~ 0.7 mm from the edge of the 3^rd^ ventricle on both sides. Activated microglia were counted in the same areas and expressed as a percent of total number of microglia. Sections from different groups (n = 4/group) were always assayed in parallel and the same camera, laser and detection settings were used throughout. Counting was performed by two independent observers and the average of the 2 values was used for statistical analysis.

#### Plasma, heart and brain cytokines

Plasma and tissue IL-1α, IL-1β, IL-2, IL-6, IL-10 and TNF-α levels were measured by Bio-Plex multiplexed magnetic bead-based immunoassay (Bio-Rad Laboratories (Canada) Ltd, Missisauga, ON, Canada) as described recently [11]. Briefly, 1:4 diluted plasma samples were incubated with coupled beads (Bio-Plex Pro rat cytokine). After a series of washes, the analyte was captured by detection biotinylated antibodies, followed by incubation with streptavidin-phycoerythrin.

The LV of the heart was separated into non-infarct (3-4mm from infarct area) and infarct area and 30-50mg of the non-infarct area was homogenized in 500μl ice-cold protein lysis buffer (Bio-Plex Cell Lysis kit: #171–304121.) containing protease inhibitor cocktails factor 1,2 (#171–304122. Bio-Rad) and 2mM PMSF (Sigma Canada Co. Oakville, ON, Canada). Brain punches (PVN and PFC) were homogenized using the same lysis buffer. The homogenate was centrifuged at 4500g for 20 min at 4° C and the supernatant was collected. Individual tissue samples with 20 μg protein were assayed for IL-1α, IL-1β, IL-2, IL-6, IL-10 and TNF-α. Samples (plasma and tissue) were processed using the Bio-Plex MAGPIX Multiplex Reader. The levels of cytokines were analyzed by a Bio-Plex Manager version 6.1 software and a pre-set external standard. Cytokine levels are expressed as pg/ml for plasma and pg/mg for brain and heart tissues.

#### Statistical analysis

Values are presented as mean ± SE. One-way ANOVA followed by a Tukey post hoc test for multiple comparisons was used for analysis of differences in one outcome measure. Two-way repeated measures ANOVA followed by a Tukey post hoc analysis was used for the cued FCT data and MWM spatial learning. Analysis of co-variance was used to evaluate the relationship of cardiac function to MI size. P ≤ 0.05 was considered statistically significant.

## Results

### Cardiac dysfunction post MI

**Fig 2:**
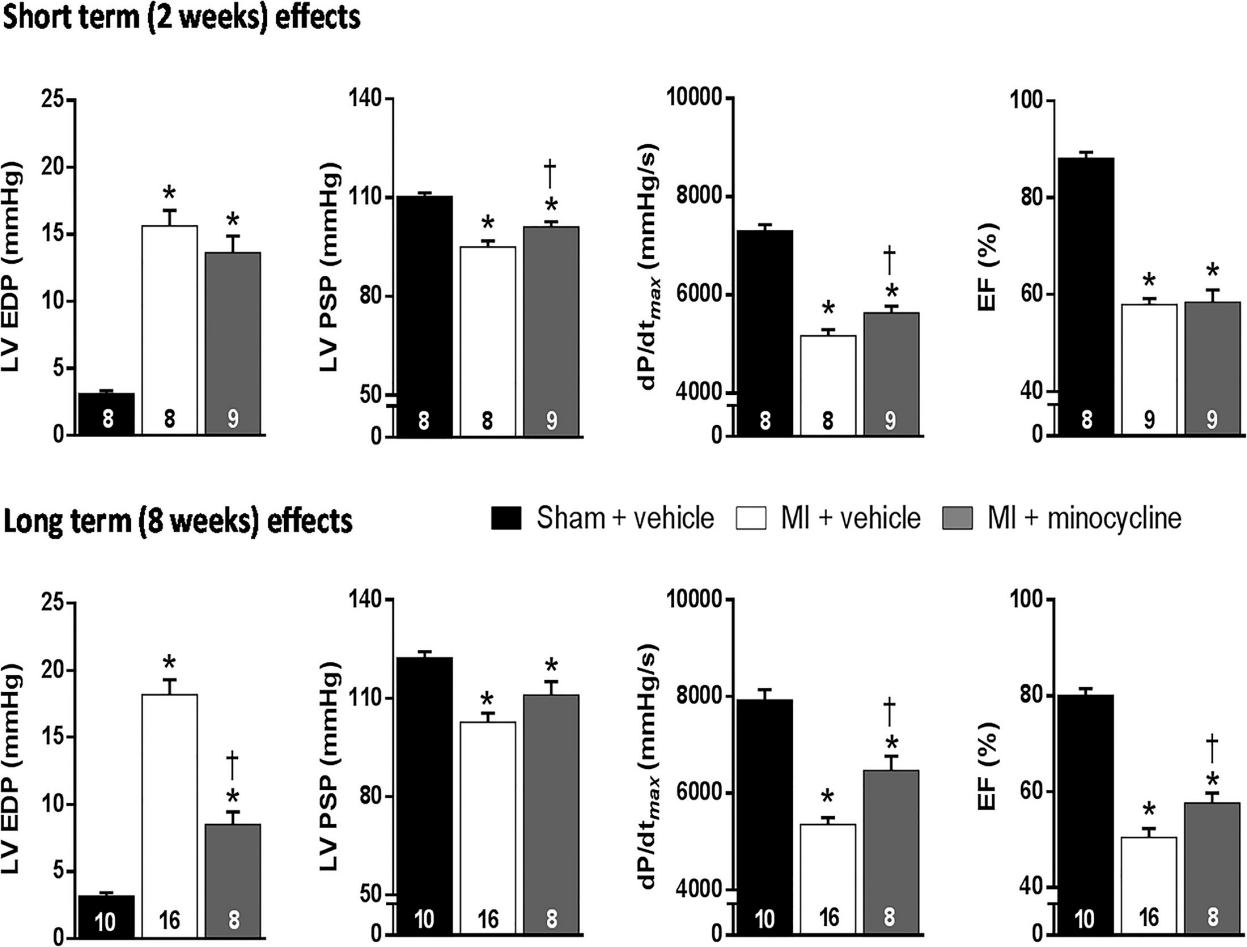
Chronic oral treatment with minocycline improves cardiac function at 2 and 8 weeks post MI. Values are means ± SE. For n/group, see bottom of bars. One-way ANOVA at 2 weeks: EDP F = 21.3, p<0.001; PSP F = 22.2, p<0.001; dP/dt _max_ F = 74.1, p<0.001 and EF F = 86.9, p<0.001. One-way ANOVA at 8 weeks: EDP F = 64.4, p<0.001; PSP F = 12.5, p<0.001; dP/dt _max_ F = 45.7, p<0.001 and EF F = 66.4, p<0.001. *p≤0.05 vs sham group, ^**†**^ p≤0.05 vs MI + vehicle.

Rats treated with vehicle post MI had average MI scar sizes of 27–29%, associated with clear evidence for cardiac dysfunction. LVEDP was markedly increased and LVPSP, dP/dt _max_ and EF decreased at both 2 and 8 weeks (Fig 2).

LV weight was modestly and RV weight more clearly increased. Oral treatment with minocycline for 2 or 8 weeks did not affect body weight. (Table 1).

**Table 1:** Effects of chronic treatment with minocycline on MI size, cardiac dimensions, LV and RV weights at 2 and 8 weeks post MI.

	Sham	MI + Vehicle	MI + Minocycline
**2 weeks Post MI**			
**MI size (%)**	**-**	**27 ± 1.0**	**24 ± 1.5**
**LV Sys. Vol. (μl/100g BW)**	**8 ± 0.6**	50 ± 3.9*	41 ± 5.5*
**LV Diast. Vol. (μl/100g BW)**	**72 ± 3.4**	120 ± 9.4*	100 ± 9.8*
**LV weight (mg/100g BW)**	**186 ± 6.3**	212 ± 3.3*	210 ± 3.0*
**RV weight (mg/100g BW)**	**46 ± 2.6**	72 ± 9.9*	**50 ± 2.2** †
**BW (g)**	**403 ± 11**	**376 ± 13**	**404 ± 10**
**8 Weeks post MI**			
**MI size (%)**	**-**	**29 ± 1.2**	**23 ± 1.3** †
**LV Sys. Vol. (μl/100g BW)**	**12 ± 1.2**	55 ± 3.9*	48 ± 5.4*
**LV Diast. Vol. (μl/100g BW)**	**63 ± 3.9**	109 ± 5.5*	113 ± 8.4*
**LV weight (mg/100g BW)**	**182 ± 6.8**	209 ± 9.7*	**183 ± 4.3** †
**RV weight (mg/100g BW)**	**41 ± 2.2**	69 ± 5.5*	**47 ± 1.6** †
**BW (g)**	**602 ± 24**	**577 ± 17**	**635 ± 11**

Values are means ± SEM (n = 8-9/group for 2 weeks. n = 8-16/group for 8 weeks). Student t-test for MI size at 2 weeks t = 2.0, p = 0.06, and at 8 weeks t = 3.0, p = 0.006

One-way ANOVA for LV syst volume at 2 weeks F = 27.9, p<0.001, and at 8 weeks F = 34.9, p<0.001

For LV diast volume at 2 weeks F = 8.1, p = 0.002 and at 8 weeks F = 19.7, p<0.001

For LV weight at 2 weeks F = 10.1, p = 0.002 and 8 weeks F = 3.3, p = 0.05

For RV weight at 2 weeks F = 6.3, p = 0.01 and at 8 weeks F = 11.2, p<0.001

For body weight, not significant at both time-points

* p*≤*0.05 vs. Sham,

**†** p*≤*0.05 vs. MI+Vehicle

At both time-points, MI scar size for the MI+ minocycline groups was in the 23–24% range, significantly lower than in the MI + Veh group at 8 weeks. At the 2-week time-point, cardiac dysfunction was modestly improved, significant for LVPSP, dP/dt _max_ and RV weight. In contrast, at 8 weeks post MI LV function was clearly improved with only a modest increase in LVEDP and significant improvements in dP/dt _max_ and EF, as well as LV and RV weight (Table 1).

**Fig 3:**
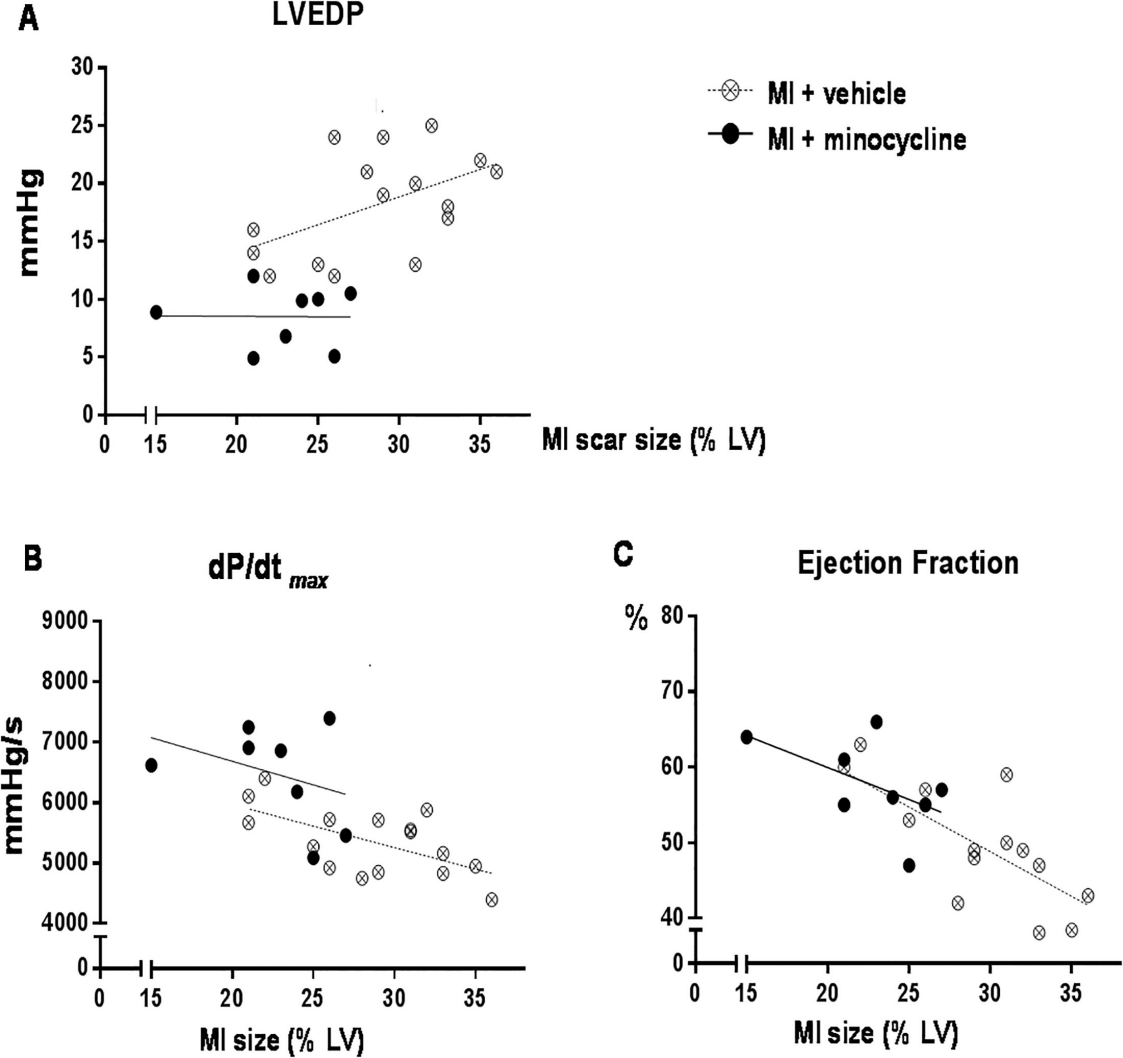
Chronic oral treatment with minocycline improves parameters of cardiac function relative to MI scar size in rats treated for 8 weeks post MI. Minocycline lowers LVEDP (A) and increases dP/dt _max_ (B) relative to MI scar size, but increases ejection fraction (C) in parallel with decrease in MI scar size. Data were analyzed by Analysis of Co-variance.

Regression analysis of MI scar size vs parameters of LV function suggests that the smaller MI size is primarily responsible for the improvement in EF, whereas LVEDP and dP/dt _max_ are also improved at similar MI scar size (Fig 3).

### Behavioural changes post MI

**Fig 4:**
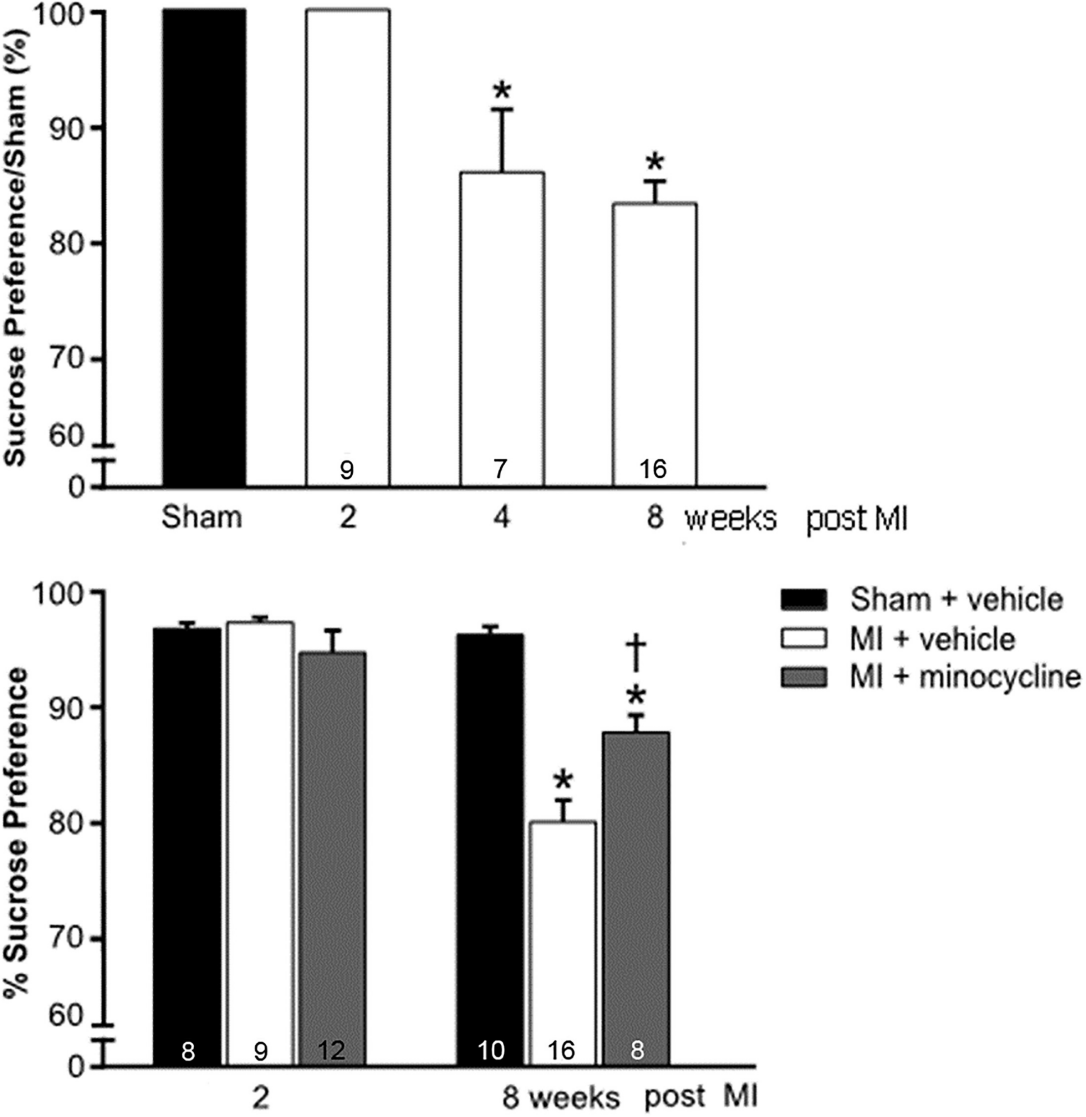
Changes in sucrose preference as percent of sham at 2, 4 and 8 weeks post MI (top panel), and improvement in the decrease in sucrose preference by minocycline at 8 weeks post MI (bottom panel). Values are means ± SE. For n/group, see bottom of bars. One-way ANOVA for time-course: F = 13.6, p<0.001. *p<0.05 vs sham or 2 weeks post MI. One-way ANOVA for 2 weeks minocycline F = 1.4, NS, and for 8 weeks F = 25.3, p<0.001. *p<0.05 vs sham ^**†**^ p<0.05 vs MI + vehicle.

Time-course for Depression-like Behaviour: At 2 weeks post MI, the SPT was still normal compared to the sham-group. By 4 weeks post MI there was a significant decrease in sucrose preference, which persisted at 8 weeks post MI (Fig 4).

**Fig 5:**
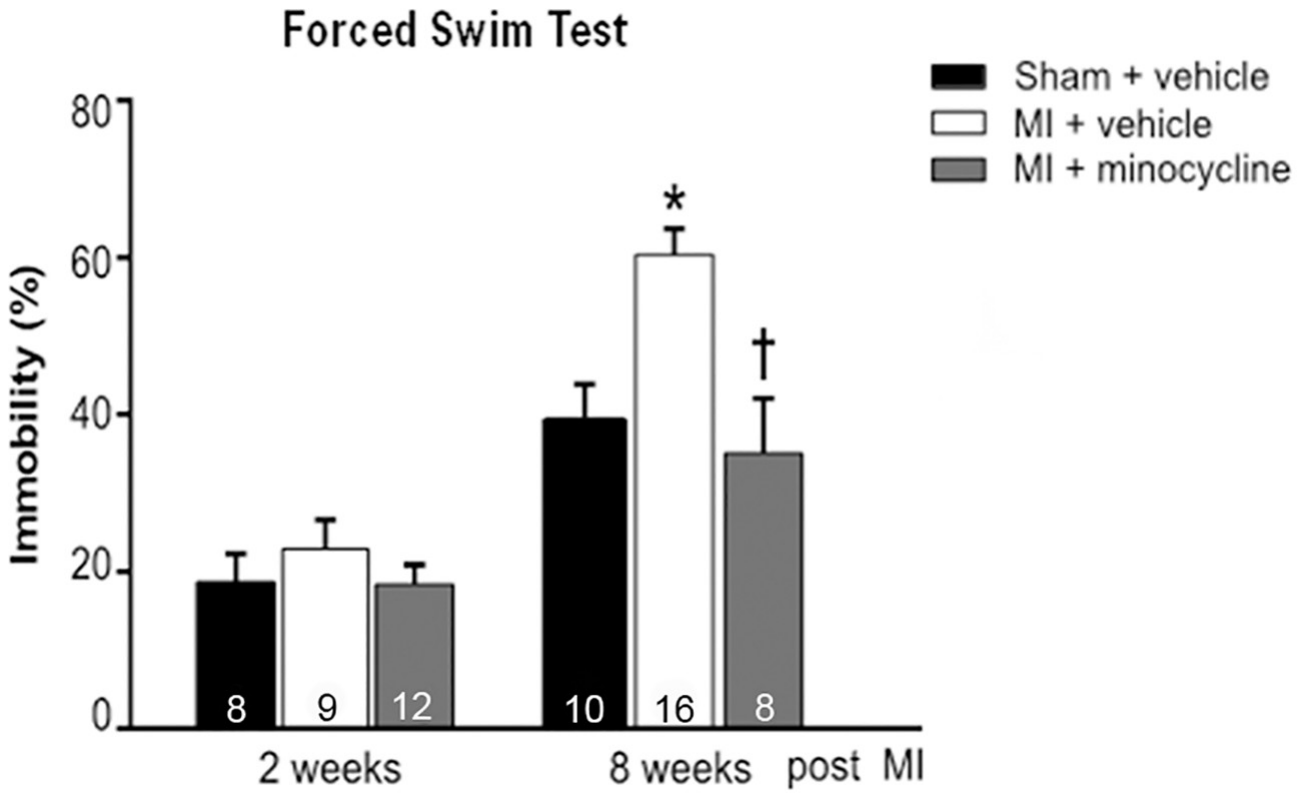
Chronic treatment with minocycline prevents the increase in immobility time in the forced swim test (FST) at 8 weeks post MI. Values are means ± SE. For n/group, see bottom of bars. One-way ANOVA at 2 weeks F = 0.7, not significant. One-way ANOVA at 8 weeks F = 9.7, p<0.001. * p<0.05 vs sham ^**†**^ p<0.05 vs MI + vehicle.

The FST followed a similar pattern with more immobility at 8 weeks post MI which is indicative of despair-type behaviour that occurs with depression (Fig 5).

Overall locomotor activity as reflected in the total distance swam and the swimming speed in the MWM and activity level in the FC chamber prior to any shock during habituation was not affected (Table 2).

**Table 2:** General locomotor activity and exploratory behaviour are not affected in rats with moderate HF at 8 weeks post MI.

**Locomotor activity in MWM test**				
	**Total distance traveled** **(cm)**		**Average swimming speed** **(cm/sec)**	
**Groups**	**Probe 1**	**Probe 2**	**Probe 1**	**Probe 2**
**Sham** **(n = 10)**	**1614 ± 71**	**1631 ± 70**	**27 ± 1.2**	**27 ± 1.1**
**MI+Vehicle (n = 9)**	**1636 ± 41**	**1643 ± 34**	**27 ± 0.7**	**27 ± 0.6**
**MI+minocycline** **(n = 8)**	**1637 ± 78**	**1451 ± 49**	**27 ± 1.3**	**24 ± 0.8**
**Interest in environment on day 1 of FCT**				
**Groups**	**Total distance moved** **(cm)**		**Exploration** **(% of habituation time)**	
**Sham** **(n = 10)**	**576 ± 35**		**93 ± 0.9**	
**MI+Vehicle (n = 9)**	**691 ± 31**		**92 ± 1.7**	
**MI+minocycline** **(n = 8)**	**684 ± 39**		**93 ± 1.1**	

Values are means ± SE

One-Way ANOVA, all not significant.

**Fig 6:**
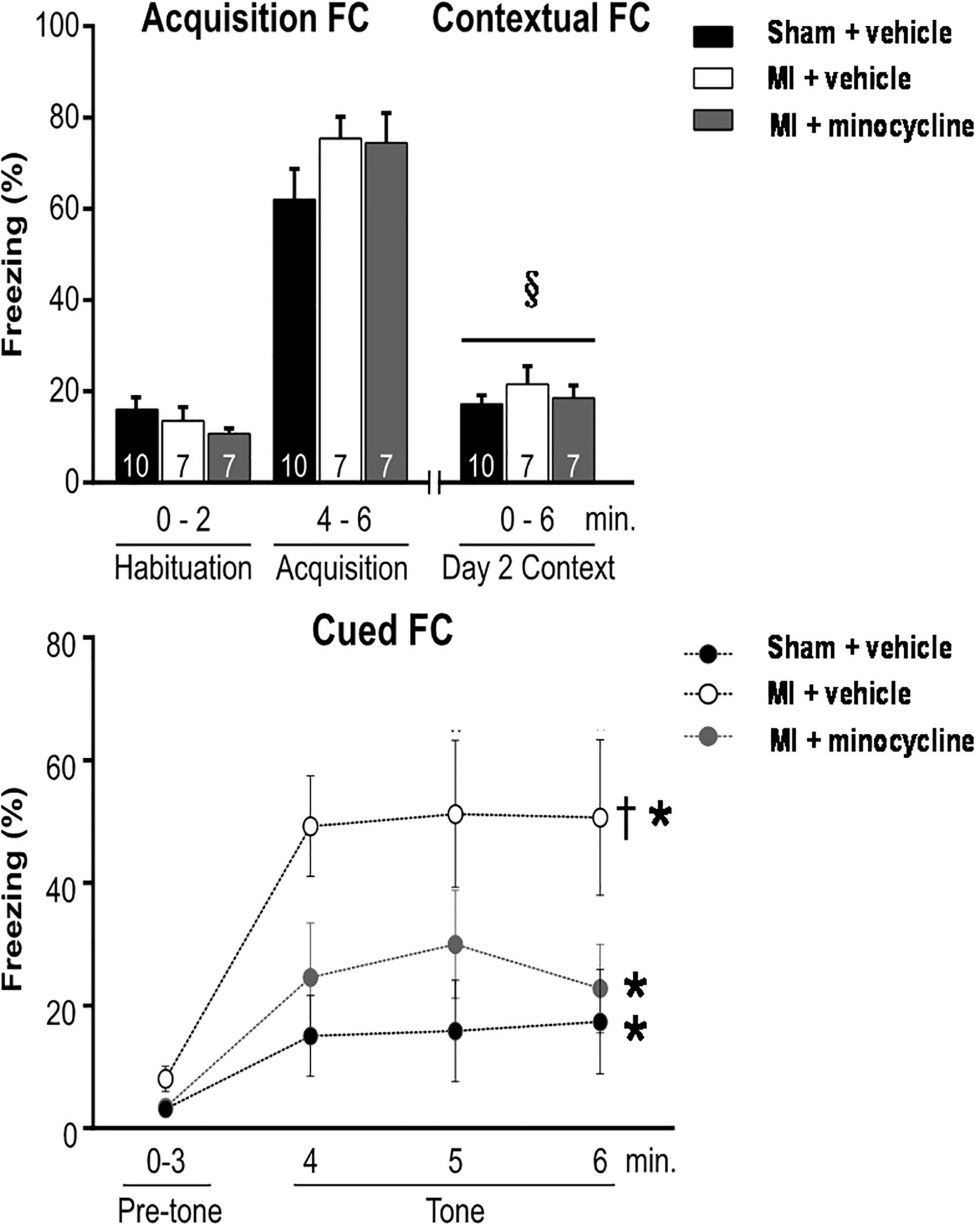
Extent of freezing during the acquisition FC on day 1, contextual FC on day 2, and the cued FC on day 3. Chronic treatment with minocycline prevents enhanced fear memory at 8 weeks post MI. Values are means ± SE. For n/group, see bottom of bars. Two-way repeated measures ANOVA for contextual FC: treatment effect F = 1.2, p = 0.33, time-effect F = 8.1, p = 0.006 and treatment × time F = 1.3, p = 0.28. § p <0.05 vs habituation day 1. Two-way repeated measures ANOVA for cued FC: treatment effect F = 4.7, p = 0.02, time-effect F = 17.6, p<0.001, and treatment × time F = 2.1, p = 0.06. *p<0.05 vs pre-tone, ^**†**^ p<0.05 vs other groups.

Freezing in the acquisition FC and context FC were similar in the sham and MI groups at 8 weeks. In contrast, freezing in the cued FC was 3-fold higher in the MI group indicating that fear associated memory was markedly enhanced (Fig 6).

**Fig 7:**
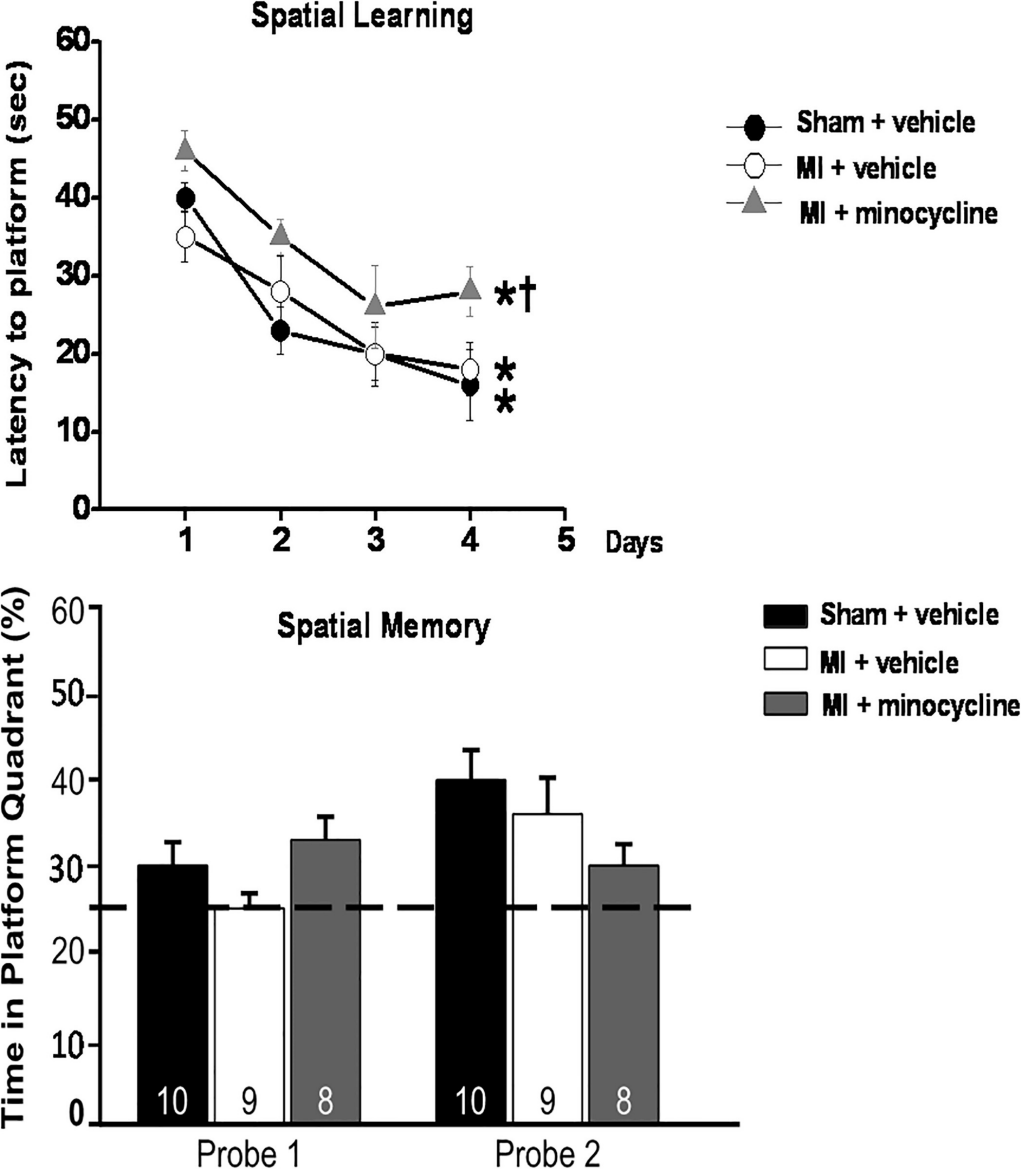
Spatial learning (top panel) and memory (bottom panel) in the MWM test are not significantly affected by HF at 7–8 weeks post MI. Chronic treatment with minocycline increases latency to platform (spatial learning), but does not significantly affect spatial memory. The 25% line in bottom panel indicates level of random exploration. Values are means ± SE. For n/group, see bottom of bars. Two-way repeated measures ANOVA for latency to platform: time-effect F = 27.5, *p<0.001 vs day 1, and treatment groups F = 5.2, ^†^ p<0.05 vs other groups. One-way ANOVA probe 1 F = 2.3, p = 0.12, and probe 2 F = 2.2, p = 0.13.

On the other hand, spatial learning and memory as asessed by the MWM was similar in the 2 groups (Fig 7).

Altogether, these findings indicate that prominent depression-like behaviour becomes apparent following the development of HF in rats post MI, whereas cognitive function is not impaired.

Effects of Minocycline: Two weeks of treatment did not affect the SPT and FST results of the MI rats. However, chronic treatment of MI rats significantly reduced immobility in the FST to the level of the sham rats (Fig 5). Sucrose preference also significantly improved with minocycline, but remained lower than in sham rats (Fig 4). In the FCT, acquisition and contextual FC outcome measures were not affected by minocycline treatment. However, the increased freezing by MI rats in the cued FC was largely prevented by minocycline (Fig 6).

In the MWM, all 3 groups showed a similar significant decline in latency to the platform over the 4 days of training, but the latency was from day 1 higher in the MI+minocycline group. On the other hand, the 3 groups showed only minor, non-significant differences for the time in platform in probe 1 and 2 (Fig 7) indicating that spatial memory did not differ.

#### Inflammatory responses

Peripheral: At 2 weeks after surgery, plasma 1L-1β and 1L-6 were non-significantly higher in rats that underwent sham-surgery compared to naive rats, but showed significant increases in rats treated post MI with vehicle (Table 3). Treatment with minocycline prevented most of these increases. At 8 weeks post MI, plasma IL-6 and TNF-α were significantly increased. Minocycline prevented the increase in plasma IL-6 (Table 3). These results indicate some pro-inflammatory effect of the surgery per se with a further increase by MI that is partly prevented by minocycline.

**Table 3:** Chronic oral treatment with minocycline prevents increases in plasma pro-inflammatory cytokines at 2 and 8 weeks post MI.

	naive (n = 4)	Sham (n = 6)	MI + Vehicle (n = 5)	MI + Minocycline (n = 5)
**2 weeks (pg/ml)**				
**IL-1α**	**94±8**	**131±20**	**129±18**	**100±17**
**IL-1β**	**97±10**	**225±27**	**298±66** ^¶^	**147±26**^†^
**IL-2**	**172±12**	**199±38**	**206±26**	**202± 57**
**IL-6**	**274±35**	**428±33**	**488±51** ^¶^	**318± 43**^†^
**TNF-α**	**275±13**	**281±22**	**255±14**	**272± 21**
**IL-10**	**52 ±7**	**62±8**	**70±6**	**67± 13**
**8 weeks (pg/ml)**		**(n = 7)**	**(n = 7)**	**(n = 8)**
**IL-1α**		**87±7**	**99±4**	**92±7**
**IL-1β**		**74±9**	**82±8**	**62±4**
**IL-2**		**167±10**	**176±4**	**158±9**
**IL-6)**		**383±17**	**467±36** **	**396±10**
**TNF-α**		**357±23**	**410±6** *	398±10*
**IL-10**		**158±15**	**178±5**	**159±7**

Values are means ± SE

One-way ANOVA for plasma at 2 weeks, IL-1β: F = 4.7, p = 0.02, and for plasma IL-6: F = 4.8, p = 0.02.

One-way ANOVA for plasma at 8 weeks, IL-6: F = 4.0, p = 0.04, and for plasma TNF-α: F = 3.9, p = 0.04.

^¶^ p<0.05 vs. naïve,

*p<0.05 vs. Sham,

^**†**^ p = 0.07 vs. MI+ Veh.

**p<0.05 vs. others

In the non-infarct area of the LV, no changes were noted at both 2 and 8 weeks post MI+veh or minocycline (Table 4).

**Table 4:** Cytokine levels are unchanged in the non-infarct area at 8 weeks post MI.

	Sham (n = 7)	MI + Vehicle (n = 7)	MI + Minocycline (n = 8)
**Non-infarct** **(pg/mg)**			
**IL-1α**	**19±1**	**18±2**	**16±2**
**IL-1β**	**44±1**	**47±3**	**43±3**
**IL-2**	**41±3**	**47±4**	**37±2**
**IL-6**	**283±9**	**255±33**	**265±35**
**TNF-α**	**54±4**	**55±7**	**50±5**
**IL-10**	**415±18**	**401±23**	**386±42**

Values are means ± SE

**Fig 8:**
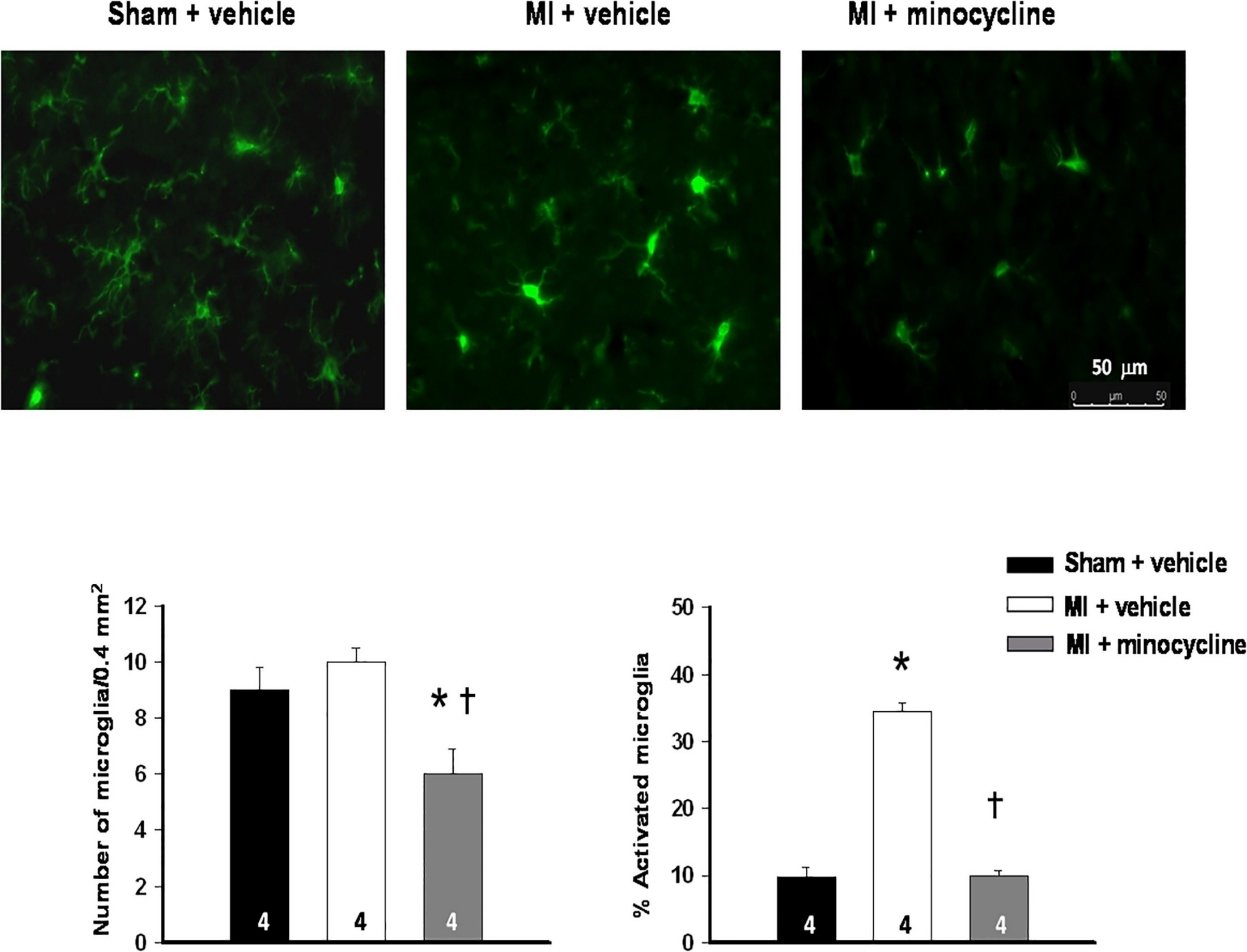
Minocycline prevents the marked microglia activation in the PVN at 8 weeks post MI. Upper panel presents representative images showing activated microglia with stronger staining, enlarged soma, and shorter, stubbier processes in the PVN of a MI + veh rat. Lower panel shows the summary data in bar graphs. Values are means ± SE (n = 4/group). One-way ANOVA for number of microglia F = 11.5, p<0.01 and for percent activated microglia F = 171.3, p<0.0001. * p<0.05 vs sham ^**†**^ p<0.05 vs MI + vehicle.

Brain: At both 2 and 8 weeks after MI, the total number of microglia was unchanged in the PVN. At 2 weeks, the percent activated microglia was 2-fold higher in the MI + vehicle group (16±1% vs 7 ±1% in sham group), but not in the MI + minocycline group (11±1%: F = 18.2, p<0.01). At 8 weeks a marked increase in the percent activated microglia was apparent in the PVN in the MI + vehicle group (Fig 8).

Treatment with minocycline fully prevented this increase. The total number of visible microglia in the PVN was also decreased (Fig 8). This enhanced microglia activation was at 8 weeks post MI associated with significant increases in IL-1⍺ and IL-2 levels in the PVN, and in IL-1⍺, IL-6, TNF-⍺ and IL-10 levels in the PFC. Minocycline prevented these increases in both the PVN and the PFC (Table 5).

**Table 5:** Chronic oral treatment with minocycline prevents increases in pro-inflammatory cytokines in the PVN and PFC at 8 weeks post MI.

	Sham (n = 11)	MI + Vehicle (n = 12)	MI + Minocycline (n = 8)
**(pg/mg)** **PVN**			
**IL-1α**	**15±1**	**29±5** **	**11±2**
**IL-1β**	**33±4**	**39±2**	**32±4**
**IL-2**	**58±6**	**86±9** **	**55±8**
**IL-6**	**244±43**	**256±16**	**215±8**
**TNF-α**	**127±14**	**135±6**	**116±4**
**IL-10**	**233±21**	**267±25**	154±3*^†^
**PFC**			
**IL-1α**	**13±1**	**16±1** **	**10±1**
**IL-1β**	**49±4**	**53±2**	**53±2**
**IL-2**	**41±2**	**46±3**	**42±2**
**IL-6**	**310±24**	389±31**	**268±25**
**TNF-α**	**197±13**	235±11*	**204±2**
**IL-10**	**193±12**	233±14**	**167±8**

Values are means ± SE

One-way ANOVA for PVN, IL-α: F = 7.1, p = 0.004; for IL-2, F = 4.9, p = 0.02; and

for IL-10, F = 6.8, p = 0.004

One-way ANOVA for PFC, IL-α: F = 8.7, p = 0.002; for IL-6, F = 4.7, p = 0.02;

for TNF-α: F = 3.8, p = 0.04; and for IL-10, F = 6.2, p = 0.006

* p<0.05 vs. Sham,

** p <0.05 vs. Sham or MI+Mino,

^**†**^ p <0.05 vs. MI+Veh.

## Discussion

The results of the present study provide new insights into the role that inflammation in the brain plays in the progression of HF and development of depression-like behaviour post MI. First, the time-course of changes indicates that depression has a delayed onset compared to HF. Cardiac dysfunction is already pronounced at 2 weeks post MI, whereas microglia activation, increases in brain cytokines and depression-like behaviour become apparent by 4–8 weeks post MI. Secondly, oral minocycline prevents increases in plasma cytokines and neuroinflammation, improves cardiac function over time, and markedly improves depression-like behaviour.

### (Neuro-) inflammation and progression of HF post MI

In the past decade, it has become apparent that increased production of cytokines in hypothalamic nuclei plays a pivotal role in sympatho-excitation and progressive cardiac dysfunction post MI [15,17]. Plasma Ang II shows a gradual increase post MI [18] as do plasma PICs (present study) [19,39]. Both may contribute to the increase in activated microglia cells and cytokines noted in the PVN of rats with HF post MI. Similar microglia activation was reported by Dworak et al [24]. Oral minocycline fully prevents both the short-term (2 weeks) and the more long-term (8 weeks) microglia activation post MI. Minocycline also prevented the increases in IL-1α and IL-2 in the PVN. This blockade of neuroinflammation may represent a direct central action of minocycline since central infusions at much lower doses also inhibit microglia activation by sc infusion of Ang II [23] or post MI [25]. However, oral treatment with minocycline also prevents the increase in 2 important plasma PICs, IL-1β and IL-6. Further studies with central infusions will be needed to ascertain whether central actions *per se* or peripheral anti-inflammatory actions of minocycline lead to the prevention of an increase in plasma PICs. The specific role of the increase in plasma PICs post MI to microglia activation and neuro-inflammation can be evaluated by peripheral infusion of eg anti-TNF-α antibodies such as infliximab [40].

Irrespective of the actual mechanisms, minocycline is rather effective in attenuating the progressive cardiac remodeling and dysfunction. Chronic treatment significantly inhibited MI scar size, prevented increases in LV and RV weight, markedly lowered LVEDP and improved parameters of systolic function. The latter appears to be related to both a smaller MI scar size and better function at similar MI scar size (Fig 3). There is extensive evidence for an important role of peripheral inflammation in the early phase of cardiac remodeling and dysfunction post MI [41, 42]. This inflammatory response may reflect enhanced myocardial synthesis of cytokines as well as a more generalized inflammatory process. Brain mechanisms contribute to the peripheral inflammation and progressive cardiac remodeling since central infusion of a mineralocorticoid receptor blocker prevents sympatho-excitation post MI, [43] shortens the cardiac inflammatory response post MI, decreases myocyte apoptosis [44] and decreases cardiac remodeling and dysfunction [43]. One may speculate that minocycline by inhibiting microglia activation and sympatho-excitation [22, 23] exerts similar cardiac effects, which may be further enhanced by possible peripheral anti-inflammatory effects. Altogether, these effects of minocycline may lead to attenuation of infarct expansion and improved contractile performance of the non-infarcted mycocardium. Further studies need to address the specific cardiac structural changes and cellular pathways which contribute to these cardiac effects of minocycline.

### (Neuro-) inflammation and depression in HF

Consistent with previous studies in rodents, [7–10] in the present study after induction of an MI and resulting HF, rats developed depression-like behaviour, ie decreased sucrose intake as a measure for anhedonia and more immobility in the forced swim test indicative of behavioural despair. Non-specific sick behaviour or impaired motor activity induced by HF could contribute to these changes. However, this unlikely plays a major role since general motor activity, weight gain and total fluid intake were not affected. Moreover, rats with the same degree of moderate HF ran on a treadmill similarly to sham-rats [45]. In agreement with depression being characterized by a blunted response to positive information, and heightened response to negative information, [46] we found enhanced fear-associated memory in the cue-associated fear condition and a lack of effect of HF on the contextual FC and spatial learning and memory in the MWM. These different behavioural changes likely reflect region-specific changes in the brain: depression-like behavior originating in eg the prefrontal cortex and nucleus accumbens, [47] enhanced fear memory from the amygdala, [47] and normal spatial memory indicative of normal cognitive function through the hippocampus. Depression is a known contributor to cognitive decline in humans, but this is mainly apparent in older subjects, [48] and young rodents may require therefore longer followup for this to become apparent [49].

There is extensive direct and indirect evidence in humans and experimental models that neuroinflammation plays a major role in the pathophysiology of depression per se [12, 13], as well as depression associated with major clinical disorders such as cancer, auto-immune diseases and diabetes [50, 51]. As discussed above, peripheral inflammation is a hallmark of HF, and likely contributes to neuroinflammation. The latter clearly occurs in hypothalamic nuclei of rodents with HF post MI [52]. The present study shows that neuroinflammation also occurs in “higher“centres such as the prefrontal cortex as reflected in significant increases in IL-1α, IL-6 and TNF-a in the PFC. Treatment with the TNF-α antagonist, etanercept, or the cytokine synthesis inhibitor, pentoxifylline, prevents/reverses the depression-like behaviour [7,9] consistent with the concept that neuro-inflammation contributes to depression-like behaviour in rats with HF.

The present study is the first study reporting the effects of minocycline on cardiac function, neuro-inflammation and behaviour in rats post MI. By 2 weeks post MI, moderate HF is present with modest microglia activation and minor changes in behaviour. Minocycline treatment prevents microglia activation and increases in plasma PICs and causes some improvement in cardiac function. Our findings suggest that (neuro) inflammation already contributes to cardiac dysfunction at 2 weeks but is not yet sufficient to induce depression-like behaviour. By 8 weeks post MI, modest further worsening of HF is apparent with marked microglia activation, increases in brain cytokines and clear depression-like behaviour. Eight weeks of treatment post MI with minocycline markedly improved cardiac function as discussed above, but also all components of the depression-like behaviour. The behavioural despair noted in the forced swim test and the enhanced fear-associated memory in the fear conditioning test were fully prevented by minocycline. The anhedonia in the sucrose preference test also significantly improved. Considering that minocycline fully prevents the chronic microglia activation, one may assume that as a result minocycline prevents the resulting increase in release of PICs in the brain. At the molecular level, cytokine-induced oxidative stress affects several neurotransmitter systems and neural circuits in the prefrontal cortex involved in behaviour [13]. The specific molecular mechanisms and pathways involved in HF and depression, and the protective effects of minocycline, are important to determine but are likely multiple and are beyond the scope of the present studies.

### Limitations of present study

For these “proof-of-principle” studies, treatment with minocycline was initiated 2 days before the MI to ensure adequate anti-inflammatory actions in the immediate, possibly critical, post MI period. Further studies will need to assess whether this is indeed a critical period or treatment started post MI can exert similar cardiac and behavioural effects, and whether treatment started in the chronic phase of HF can reverse depression and improve cardiac function. Further studies are also needed to assess whether central anti-inflammatory and behavioural effects of oral minocycline represent direct central actions or indirect responses to peripheral anti-inflammatory actions and improved cardiac function.

### Clinical perspective

The present studies in a well-established experimental model for HF in humans post MI demonstrate the major role of peripheral and neuro—inflammation for both progression of HF and depression-like behaviour post MI. No effective mechanism-based or empirical treatments for depression in patients with HF are presently available. Inhibition of inflammation by minocycline or related drugs may represent such a strategy, if treatment is also effective when started post MI or in the more chronic phase of HF. Clinical trials with minocycline have been started for treatment of patients with hypertension [22]. Based on the present findings, it appears worthwhile to initiate studies with minocycline in patients early post MI to assess its role as a new therapeutic strategy to inhibit development of HF and associated depression.
